# RETRACTION: Consequences of hydrogenated vegetable fat substitution with Ajwa seed oil on physicochemical and nutritional aspects of functional cookies

**DOI:** 10.1002/fsn3.4093

**Published:** 2024-04-05

## Abstract

**RETRACTION:** Muhammad Qasim Raza, Muhammad Umair Arshad, Muhammad Sajid Arshad, Faqir Muhammad Anjum, Ali Imran, Aftab Ahmed, and Haroon Munir. “Consequences of Hydrogenated Vegetable Fat Substitution with Ajwa Seed Oil on Physicochemical and Nutritional Aspects of Functional Cookies.” *Food Science & Nutrition* 8 (2020): 1365–1374, https://doi.org/10.1002/fsn3.1383.

The above article, published online on 03 February 2020 in Wiley Online Library (wileyonlinelibrary.com), has been retracted by agreement between the journal's Editor‐in‐Chief, Y. Martin Lo, and Wiley Periodicals, LLC. The retraction has been agreed to following concerns about the peer review process. The editorial office found unambiguous evidence that the manuscript was accepted solely based on compromised and insufficient reviewer reports. This evidence was further investigated and verified by the publisher and the Editor‐in‐Chief. As a result, the conclusions of this article are not considered reliable. The authors were notified of the retraction and disagreed with this decision.

**RETRACTION:** Muhammad Qasim Raza, Muhammad Umair Arshad, Muhammad Sajid Arshad, Faqir Muhammad Anjum, Ali Imran, Aftab Ahmed, and Haroon Munir. “Consequences of Hydrogenated Vegetable Fat Substitution with Ajwa Seed Oil on Physicochemical and Nutritional Aspects of Functional Cookies.” *Food Science & Nutrition* 8 (2020): 1365–1374, https://doi.org/10.1002/fsn3.1383.

The above article, published online on 03 February 2020 in Wiley Online Library (wileyonlinelibrary.com), has been retracted by agreement between the journal's Editor‐in‐Chief, Y. Martin Lo, and Wiley Periodicals, LLC. The retraction has been agreed to following concerns about the peer review process. The editorial office found unambiguous evidence that the manuscript was accepted solely based on compromised and insufficient reviewer reports. This evidence was further investigated and verified by the publisher and the Editor‐in‐Chief. As a result, the conclusions of this article are not considered reliable. The authors were notified of the retraction and disagreed with this decision.

